# Polarization Pruning: Reliability Enhancement of Hafnia‐Based Ferroelectric Devices for Memory and Neuromorphic Computing

**DOI:** 10.1002/advs.202407729

**Published:** 2024-09-26

**Authors:** Ryun‐Han Koo, Wonjun Shin, Jangsaeng Kim, Jiyong Yim, Jonghyun Ko, Gyuweon Jung, Jiseong Im, Sung‐Ho Park, Jae‐Joon Kim, Suraj S Cheema, Daewoong Kwon, Jong‐Ho Lee

**Affiliations:** ^1^ Department of Electrical and Computer Engineering and Inter‐university Semiconductor Research Center Seoul National University Seoul 08826 Republic of Korea; ^2^ Research Laboratory of Electronics Massachusetts Institute of Technology Cambridge MA 02139 USA; ^3^ Department of Electrical Engineering Hanyang University Seoul 04763 Republic of Korea; ^4^ Ministry of Science and ICT Sejong‐si 30109 South Korea; ^5^ Department of Semiconductor Convergence Engineering Sungkyunkwan University Suwon Gyeonggi‐do 16419 Republic of Korea

**Keywords:** Ferroelectrics, field effect transistor, memory devices, neuromorphic computing, polarization

## Abstract

Ferroelectric (FE) materials are key to advancing electronic devices owing to their non‐volatile properties, rapid state‐switching abilities, and low‐energy consumption. FE‐based devices are used in logic circuits, memory‐storage devices, sensors, and in‐memory computing. However, the primary challenge in advancing the practical applications of FE‐based memory is its reliability. To address this problem, a novel polarization pruning (PP) method is proposed. The PP is designed to eliminate weakly polarized domains by applying an opposite‐sign pulse immediately after a program or erase operation. Significant improvements in the reliability of ferroelectric devices are achieved by reducing the depolarization caused by weakly polarized domains and mitigating the fluctuations in the ferroelectric dipole. These enhancements include a 25% improvement in retention, a 50% reduction in read noise, a 45% decrease in threshold voltage variation, and a 72% improvement in linearity. The proposed PP method significantly improves the memory storage efficiency and performance of neuromorphic systems.

## Graphical abstract

This study introduces Polarization Pruning (PP) to address reliability issues in hafnium‐based ferroelectric devices. PP aligns ferroelectric domains by adding a weak counterpulse after conventional pulses, enhancing retention by 20–25%, reducing read noise by 50%, variability by 45%, and nonlinearity by 72%. This advancement significantly benefits nonvolatile memory and AI hardware, offering novel a solution for more reliable ferroelectric devices.

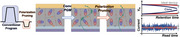

## 1 Introduction

The advancement of future electronics beyond Moore's paradigm requires the introduction of novel materials that exploit diverse functionalities and methods to control them at different scalable dimensions.^[^ ^1^, ^2^ ^]^ Ferroelectricity, which is characterized by the spontaneous polarization of materials that can be controlled by an external electric (*E*)‐field is a promising avenue for such exploration.^[^ ^3^, ^4^, ^5^, ^6^, ^7^, ^8^, ^9^, ^10^, ^11^, ^12^, ^13^, ^14^, ^15^, ^16^, ^17^, ^18^, ^19^, ^20^, ^21^, ^22^, ^23^, ^24^ ^]^ Of particular interest is the emergence of hafnia‐based ferroelectrics, which exhibit a wide array of applications, including non‐volatile memory,^[^ ^6^, ^7^, ^8^, ^9^, ^10^, ^11^, ^12^, ^13^ ^]^ negative capacitance,^[^ ^14^ ^]^ and energy storage.^[^ ^15^ ^]^ The discovery of ferroelectricity in hafnia‐based oxides in 2011 marked a significant milestone and triggered a surge in research efforts.^[^ ^25^, ^26^ ^]^ Notably, in contrast to perovskite oxides, hafnia has been a pivotal component of high‐K‐metal‐gate (HKMG) technology since the mid‐2000s, and its compatibility with modern complementary metal—oxide–semiconductor (CMOS) technology holds tremendous promise for high‐volume semiconductor manufacturing across a range of commercial products.^[^ ^25^, ^26^ ^]^

The integration of ferroelectricity with CMOS technology offers an opportunity to substantially reshape the landscape of electronic devices and challenge the prevailing computing paradigm rooted in the von Neumann architecture.^[^ ^27^, ^28^ ^]^ It is characterized by a distinct segregation between memory blocks and logic cores, which currently faces challenges in efficiently processing vast volumes of data with high throughput and energy efficiency for artificial intelligence (AI) applications. Integrating ferroelectric (FE) materials with CMOS technology broadens the available material options and enriches the understanding of the underlying physics of devices. This integration is crucial for the development of dual‐function elements that can simultaneously perform memory storage and computation tasks, thereby supporting more complex, data‐intensive AI applications, such as in‐memory computing and neuromorphic computing. Such elements can play a pivotal role in data‐centric AI applications by offering potential solutions to circumvent the constraints imposed by the von Neumann bottleneck.^[^ ^10^, ^25^, ^26^, ^29^ ^]^

The path forward for FE electronics in the domain of data‐centric applications is inevitably accompanied by reliability problems, including poor field‐cycling endurance,^[^ ^30^, ^31^ ^]^ significant read noise,^[^ ^32^, ^33^ ^]^ large variability,^[^ ^34^, ^35^ ^]^ and poor retention.^[^ ^31^, ^36^ ^]^ Among these problems, poor endurance characteristics are considered to be the most critical in FE devices. Repeated program (PGM) and erase (ERS) operations in FE devices lead to changes in the memory window owing to wake‐up, aging, imprinting, and fatigue effects, ultimately resulting in memory window collapse. Research has revealed that the primary cause of poor endurance is interfacial issues rather than inherent deficiencies within the ferroelectric layer.^[^ ^25^, ^26^, ^30^, ^31^ ^]^ Consequently, extensive research efforts have been directed toward addressing these challenges through the meticulous engineering of gate stack designs and enhancing the interface quality via scavenging effects or optimizing the dielectric constant of the interlayers. Drawing parallels with historical developments in HKMG technology, reminiscent of the early stages of its evolution, provides valuable insights into potential strategies for mitigating poor endurance characteristics. Consequently, recent studies have shown notable advancements in endurance, with reported endurance levels ranging from 10^5^ to 10^12^ cycles, surpassing those of charge trap flash memory.^[^ ^37^ ^]^

An underaddressed yet critical aspect of reliability in FE electronics is the read noise and retention degradation stemming from the inherent characteristics of the FE film. Unlike device‐to‐device and cycle‐to‐cycle variations, which can be mitigated through process refinements and additional circuitry, the device read variation (read noise), which is an intrinsic device attribute, cannot be easily reduced.^[^ ^38^, ^39^ ^]^ This noise profoundly affects both the learning and inference processes in data‐centric applications, especially as the device dimensions diminish.^[^ ^40^ ^]^ Moreover, FE materials, by their nature, introduce diverse noise sources, including phonon scattering and additional carrier scattering owing to polarization domain reversal, exhibiting larger noise profiles compared to traditional silicon channel materials.^[^ ^41^, ^42^, ^43^ ^]^ Retention degradation also occurs because of the inherent properties of ferroelectric domains, which are influenced by interactions with various defects and ions within the film, such as oxygen vacancies. Owing to these defects, the ferroelectric domains are not fully aligned in the desired direction, and their retention is degraded by changing the depolarization field.^[^ ^31^, ^36^ ^]^ Such retention degradation distorts the optimized synaptic weight distribution during the vector‐matrix multiplications, degrading the system accuracy.^[^ ^44^, ^45^ ^]^ Furthermore, retention and endurance characteristics are closely intertwined in a tradeoff relationship. Specifically, weaker (stronger) PGM/ERS pulse conditions improve (deteriorate) the endurance but deteriorate (improve) the retention characteristics. Therefore, improving the retention characteristics without deteriorating the endurance provides additional operational headroom under FE‐based operating conditions, potentially leading to overall improvements in reliability. Therefore, it is imperative to devise methodologies to improve noise and retention.^[^ ^46^, ^47^ ^]^ Moreover, the requirement of an extensive degree of integration in data‐centric applications underscores the necessity for elucidating these methodologies at the array level rather than at the individual component level.

In this study, we propose and demonstrate a new operational protocol called polarization pruning (PP) to address reliability problems in hafnia‐based ferroelectrics for data‐centric applications. The PP is designed to enhance both read‐noise reduction and retention characteristics without compromising endurance. This is achieved by aligning the ferroelectric domains in the desired direction after program (PGM) and erase (ERS) operations. Alignment is enabled by applying a pulse of the opposite sign to the preceding PGM or ERS pulse, but with a reduced magnitude. To examine the efficacy of the PP method and optimize the PP pulse scheme, a modified version of the positive–up–negative–down (PUND) measurement was devised. In addition, low‐frequency noise (LFN) measurements were performed to investigate the physics underlying the reduction in read noise and retention improvement by probing the internal dynamics of the ferroelectric domains. It is demonstrated that the conductance levels are doubled and the retention characteristics are improved by more than 20 percent by PP, significantly improving the data storage density and stability for neuromorphic computing. To establish the broad applicability of the proposed PP in ferroelectric devices, its efficacy is examined across two‐terminal ferroelectric tunnel junctions (FTJs) and three‐terminal ferroelectric field‐effect transistors (FeFETs). Regardless of the device structures, reliability improvements stemming from PP are observed in both FTJs and FeFETs, thereby affirming the universality of PP. Furthermore, beyond verification at the single‐device level, empirical investigations have been extended to the array level, emphasizing the pivotal role of PP in practical data‐centric operations, notably in neuromorphic computing. The results of this study hold broad applicability across various types of ferroelectric devices, facilitating both the scientific comprehension and technological implementation of hafnia‐based data‐centric applications.

## 2 Results

### 2.1 Proposition of Polarization Pruning

**Figure 1:**
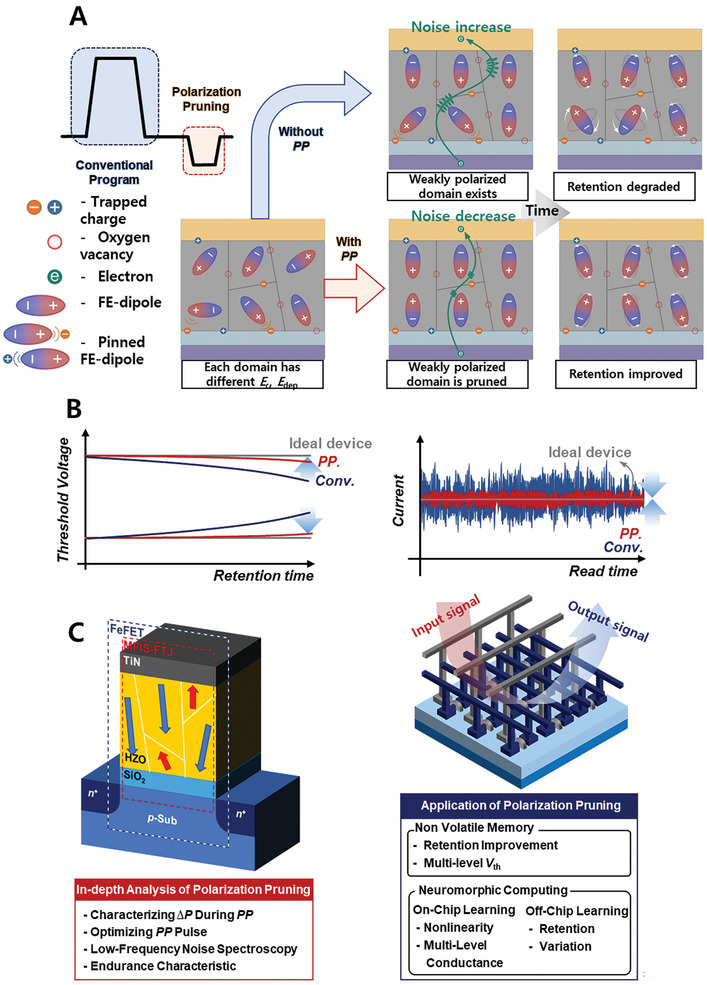
Schematic of PP mechanism and its applications. A) Schematic diagram illustrating the PP pulse scheme and the corresponding polarization state in ferroelectric materials. B) Schematic highlighting the improvements in device reliability achieved through polarization pruning. Two major enhancements: an improvement in retention characteristics (left), and a reduction in read noise (right), demonstrating the positive impact of PP on the stability and accuracy of FE‐based devices. C) Overview of FE‐based devices used in this study for analyzing the PP mechanism (left). Both FeFET and FTJ with the same gate stack structure are utilized. Additionally, the methods employed for analyzing the PP mechanism are summarized, providing a comprehensive view of the investigative approach taken. Schematic illustrating the application strategies of PP in FE‐based memory systems (right).

**Figure 2:**
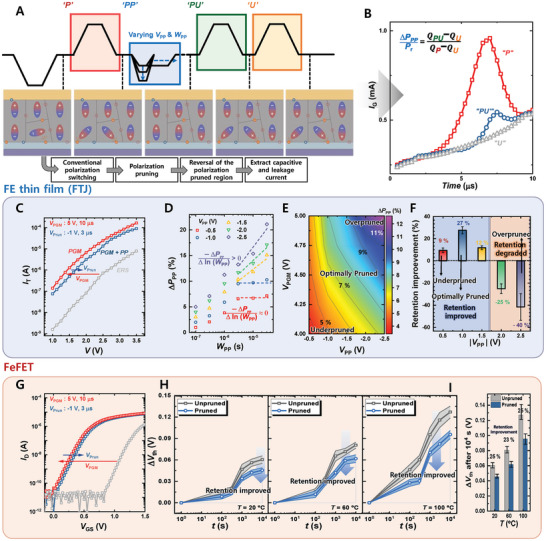
Analysis of PP mechanism and improvement of retention characteristics. A) Schematic of the modified PUND pulse scheme and the corresponding ferroelectric domain schematic for each phase. B) Measured *I_T_* during ramp time in the “P” (Positive), “PU” (Pruning‐Up), and “U” (Up) phases of the modified PUND measurement. C) *I_T_–V* characteristics of FTJs for different states. D) Relationship between pruned polarization and WPP for various VPP values. E) A contour map depicting pruned polarization as a function of VPGM and VPP, highlighting the regions of underpruned, optimally pruned, and overpruned. F) Degree of retention improvement as a function of VPP. G) VGS‐ID curves in FeFET for different states. H) Time‐dependent change in Δ*V*th in FeFET with and without the application of PP, shown at different temperatures (20, 60, and 100 °C). I) Change in ΔVth of FeFET over 1000 s at various temperatures, comparing the effects of PP application versus no pruning.

**Figure** **1**A shows a schematic of the proposed PP method. The logic behind PP can be explained as follows: In ferroelectric HfO_2_ films, each FE domain exhibits a distinct coercive electric field (*E*_c_) and depolarization field (*E*_dep_) owing to charged defects at the electrode/FE or FE/dielectric (DE) interfaces, traps at grain boundaries, and interactions between grains. Therefore, upon applying a program pulse, not all the FE dipoles align in the desired direction. These incompletely or weakly aligned FE domains compromise the reliability of the FE‐based memory operations. To address this problem, we propose a novel approach of applying a PP pulse, which carries the opposite sign of the preceding PGM or ERS pulse, but is smaller in magnitude. The PP effectively reoriented the weakly aligned FE domains in opposite directions. The right part of Figure 1A shows the changes in the FE domains over time, comparing conditions without (top) and with (bottom) implementation of the PP. Without PP, numerous weakly polarized domains are created, which rapidly become disorganized owing to their substantial *E*_c_ and *E*_dep_, resulting in a degraded retention performance and worsened carrier scattering within the FE materials. Applying a PP can improve the retention characteristics and reduce read noise by stabilizing and reorienting weakly polarized domains in advance, thereby achieving more reliable operation of the FE‐based memory. Figure 1B depicts a schematic of the advantages of the proposed PP method for memory and neuromorphic applications. Compared to the conventional PGM/ERS methods without PP, the PP method 1) significantly improves retention characteristics (left side of Figure 1B) and 2) effectively reduces read noise (right side of Figure 1b). This study not only shows the efficiency of PP but also offers a method to quantitatively analyze its impact on the two most representative types of FE‐based devices, FTJ, and FeFETs. (Figure 1C). The FTJ and FeFET are fabricated at both the single‐device and large‐array levels, demonstrating the advantages of PP in memory and neuromorphic computing with a systematic analysis of the underlying physics behind the improvement. Specifically, the advantages of PP in memory and neuromorphic computing can be categorized into three distinct aspects: 1) Memory storage efficiency: PP improves retention and reduces read noise, which is crucial for supporting a larger number of memory states and enhancing memory storage efficiency. 2) On‐chip neuromorphic system performance: PP improves the nonlinearity and increases the number of conductance levels, which plays a pivotal role in enhancing the performance of on‐chip learning‐based hardware neuromorphic systems. 3) Off‐chip neuromorphic system performance: PP improves retention and reduces variation, which plays a pivotal role in improving the performance of off‐chip learning‐based hardware neuromorphic systems.

### 2.2 Unraveling the Mechanism of Polarization Pruning

Here we demonstrate the newly proposed “Modified PUND” operation scheme, designed to quantitatively analyze the effects of PP on FE devices, as shown in **Figure** **2**A. Contrary to the conventional PUND measurement method, the difference in the current measured during the Positive (“P”) and Up (“U”) phases represents the pure displacement current value owing to polarization switching,^[^ ^48^ ^]^ this modified approach introduces additional PP pulse (“PP”) and a pruning‐up (“PU”) phase between the standard “P” and “U” phase. This enables the quantitative measurement of the polarization pruned by the PP. Further details on the functioning and measurement of the Modified PUND are provided in the Experimental Section. Based on the Modified PUND method, we analyzed the number of ferroelectric domains pruned in metal–FE—insulator–semiconductor (MFIS) structured FTJ. The fabrication process of the FTJ is illustrated in Figure S1 (Supporting Information) and in the Experimental Section.^[^ ^49^ ^]^ The transmission electron microscopy (TEM) images, X‐ray photoelectron spectroscopy, and grazing incidence X‐ray diffraction analysis results, PUND measurement results, and *P*–*V* and tunneling current (*I*_T_)–*V* curves of the MFIS‐FTJs are shown in Figure S2 (Supporting Information). Figure 2B shows Modified PUND measurements of the fabricated FTJ. Figure 2C shows the *I*_T_–*V* characteristics of the FTJs in the three states: ERS, PGM, and after applying PP after PGM. Figure 2B shows the relationship between the measured amount of ratio of pruned polarization during PP (∆*P*_PP_/*P*_r_) and the width (*W*_PP_) and amplitude (*V*_PP_) of the pruning pulse. The PGM voltage (*V*_PGM_) remains constant when only the PP pulse varies. The switching voltages of the fabricated MFIS FTJ (Figure S2D, Supporting Information), pruning pulses with amplitude of −0.5 V or −1.0 V are too small to switch FE dipoles in the opposite direction (a pulse of −4.0 V, 10 µs is required for complete opposite direction switching). Therefore, in cases where *V*_PP_ is −0.5 V or −1.0 V, a ∆*P*_PP_/∆ ln (*W*_PP_) value close to zero is expected. Indeed, without a preceding PGM pulse, a ∆*P*_PP_/∆ ln (*W*_PP_) value near zero is observed across the entire *W*_PP_ range for *V*_PP_ of −0.5 V or −1.0 V (Figure S3A, Supporting Information). However, following a PGM pulse, with *V*_PP_ at −0.5 V or −1.0 V, *W*_PP_ values below 3 µs exhibited a ∆*P*_PP_/∆ ln (*W*_PP_) greater than zero (Figure 2D). This indicates that a PP pulse that is smaller and shorter than the preceding PGM pulse effectively removes weakly polarized domains that are not fully aligned by the previous PGM pulse. For *W*_PP_ values above 3 µs, ∆*P*_PP_/∆ ln (*W*_PP_) reaches zero (saturated) as all weakly polarized domains have been pruned. When the *V*_PP_ is increased to −1.5–−2.0 V, a ∆*P*_PP_/∆ ln (*W*_PP_) value greater than zero is observed in all *W*_PP_ ranges, regardless of the existence of previous PGM pulses. This is owing to the relatively large *V*_PGM_‐induced switching of well‐aligned FE domains and not only weakly polarized domains. Therefore, by comparing the slope of ∆*P*_PP_ – *W*_PP_ with various *V*_PP_, with and without PGM pulses, we can determine the conditions under which weakly polarized domains are pruned and actual programming (switching of well‐aligned FE domains) occurs. Similarly, Figure S3B (Supporting Information) shows the effect of varying the number of PP pulse applications (*N*_PP_) on ∆*P*_PP_ while fixing the *W*_PP_ values at 3 µs. Consistent with the results in Figure 2D, for *V*_PP_ above −1.0 V, ∆*P*_PP_ saturates despite an increase in *N*_PP_, while for *V*_PP_ below −1.0 V, ∆*P*_PP_ continues to increase as *N*_PP_ increases. Therefore, when *V*_PP_ is below −1.5 V, it is named as an “overpruned” state, indicating that not only the weakly polarized domains but also the well‐aligned domains switch in the opposite direction. Conversely, for a *V*_PP_ of −0.5 V, the scenario is classified as an “underpruned” state due to insufficient pruning, compared to −1.0 V. When *V*_PP_ is −1.0 V for 3 µs, this condition is referred to as “optimally pruned” state since ∆*P*_PP_ saturates even with a single pulse application. Figure 2E shows the ratio of pruned polarization as a function of *V*_PGM_ and *V*_PP_. As *V*_PP_ increases, more pruning occurs, leading to an increase in ∆*P*_PP_. Similarly, as *V*_PGM_ increases, ∆*P*_PP_ also rises. This is because a larger *V*_PGM_ polarizes more domains, thereby increasing the number of weakly polarized domains. This contour map shows the conditions of *V*_PP_ for the underpruned, optimally pruned, and overpruned states of each *V*_PGM_.

### 2.3 Retention Improvement by Polarization Pruning

As mentioned previously, PP is designed to improve the reliability of FE devices. First, we focus on retention characteristics by preemptively reorienting weakly polarized domains that can easily switch in the opposite direction and cause retention loss. Figure 2F shows the change in polarization retention characteristics of FTJs after 1000 s compared to the unpruned case (∆*P*_r_/∆*P*_r,unpruned_) versus the magnitude of *V*_PP_. The retention characteristics were measured when they had the same magnitude of polarization for a fair comparison. The results show that compared with the conventional unpruned program method, the underpruned case exhibits a 9% improvement in retention, the optimally pruned case shows a 27% improvement, and the overpruned case exhibits a 40% degradation in retention. As expected, the underpruned case shows some improvement in the retention characteristics, but less than the optimal case, owing to the remaining weakly polarized domains. In the overpruned case, the retention characteristics were worse than those in the unpruned scenario, which is attributed to the majority of domains switching in the opposite direction, creating excessive *E*_dep_. Figure S3C (Supporting Information) shows retention characteristics over time (*t*) for unpruned, underpruned (*V*_PP_ = −0.5 V), optimally pruned (*V*_PP_ = −1.0 V), and overpruned (*V*_PP_ = −2.5 V) conditions. It is important to note that the improvement in retention by PP also applies to ERS. The trend of the ratio of the pruned polarization according to the magnitude and sign of *V*_PP_ is shown in Text 1 (Supporting Information). Figure S3E (Supporting Information) shows the retention characteristics over 1000 s in the PGM and ERS states of the FTJ. Figure S3F (Supporting Information) shows the ratio of pruned polarization as the FTJ area is downscaled, and Figure S3G (Supporting Information) shows the degree of improvement in retention upon the application of PP as the FTJ area was scaled down. PP exhibits negligible dependence on the device area, indicating its effectiveness even in highly scaled‐down FTJs.

Furthermore, the improvement in retention by PP is also applicable to more complex structures such as FeFETs. The fabrication process and the structure of the FeFETs are shown in Figure S4 (Supporting Information) and in the Experimental Section.^[^ ^50^ ^]^ Figure 2G presents the transfer curves (*I*_D_–*V*_GS_) for the ERS, PGM, and PGM after the PP states of the FeFET. A 5.0 V of 10 µs pulse is applied for the PGM, and a −4.0 V of 10 µs erase pulse (*V*_ERS_) is applied for the ERS state. For PP in the PGM state, a −1.0 V of 3 µs pulse is applied. After applying the PP pulse to the PGM, a slight positive shift in *V*_th_ is observed. Figure 2H shows the retention characteristics of the FeFET measured at different temperatures (*T*s) for 20 FeFETs, with solid lines indicating the average values and lightly shaded areas that indicate the minimum–maximum error bars. Figure 2I shows the change in *V*_th_ after 1000 s for both the pruned (PP applied) and unpruned conditions, measured at different *T*s. For a fair comparison, the retention characteristics were measured in the same *V*_th_ state. As *T* increased, the FE domain movement is activated, in which deteriorates the retention characteristics. At all *T*, a 23–25% improvement in retention characteristics was observed with pruning. Since the retention characteristics of FeFETs are influenced by factors other than the FE domain, such as charge trapping and detrapping,^[^ ^28^, ^29^ ^]^ it is necessary to verify whether the observed improvement in retention is due to enhanced polarization switching retention. Figure S5A (Supporting Information) shows the retention characteristics measured at various *T*s values for 20 FTJs, with solid lines indicating the average values and lightly shaded areas indicating the minimum–maximum error bars. Figure S5B (Supporting Information) shows the polarization losses after 1000 s for pruned and unpruned conditions at various *T*s values in the FTJs. As with the FeFET results, retention characteristics worsen with increasing temperature, and a 27–32% improvement in retention characteristics is noted upon applying polarization pruning. This is consistent with the 23–25% improvement observed in the FeFET upon applying PP (Figure 2I), indicating that the improvement in retention characteristics of FeFETs is attributed to the enhancement in polarization switching retention due to PP, not charge trapping‐detrapping effects.

Retention and endurance characteristics have a tradeoff relationship in memory devices. This is because improving retention using a high *V*_PGM_ or a long PGM time can degrade endurance characteristics. However, PP significantly increases the retention characteristics without compromising endurance. By applying a short pulse with low amplitude immediately after the PGM, the proposed method avoids the endurance deterioration observed in previous studies. Refer to Text 2 (Supporting Information) for detailed measurements of endurance characteristics.

### 2.4 Read Noise Reduction by Polarization Pruning

This work focuses on another advantage of PP: reduced read noise. LFN measurements were performed to systematically investigate the effects of PP on the read noise and gain a deeper understanding of the mechanism behind PP. LFN measurements convert the fluctuation of current in the time domain into power spectral density (PSD) in the frequency domain, enabling accurate analysis of fluctuations in various physical quantities involved in carrier transport processes.

**Figure 3:**
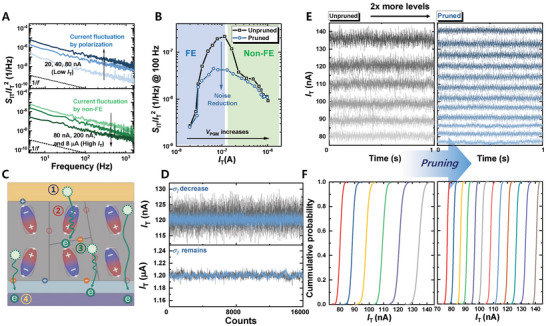
Improvement of read noise in FE‐based devices through PP. A) *S*_IT_/*I*_T_^2^ of FTJ versus different frequencies in different *I*_G_ (PGM state). B) *S*_IT_/*I*_T_^2^ values sampled at 100 Hz as a function of the *I*_T_. This graph demonstrates the reduction in read noise achieved through PP. C) A schematic explaining the mechanism of how PP reduces FE‐dipole fluctuation and consequently improves read noise. D) Results of transient read current measurements in FTJ at low current levels (120 nA, top) and high current levels (1.2 µA, bottom), comparing devices with and without PP application. The measurement frequency is 3200 Hz over a period of 5 s. E) Measurement results of transient read current in FTJ within the current range of 75 nA to 150 nA, with and without PP application. F) Cumulative probability graphs for different levels of *I*_G_, with and without PP application.

**Figure** **3**A shows the normalized PSD of *I*_T_ (*S*_IT_/*I*_T_^2^) of the FTJs for different PGM states versus the frequency. Detailed noise measurement methods is shown in the Experimental Section. The *I*_T_ varies from 20 nA to 8 µA based on the PGM states at the same read voltage. Notably, the 1/*f* noise behavior was observed across all PGM states. Interestingly, in FTJs, *S*_IT_/*I*_T_^2^ increases with larger current magnitudes but then decreases beyond a certain current level. This contrasts with other electrical devices, such as RRAM or phase‐change random‐access memory,^[^ ^51^, ^52^ ^, 53]^ where the noise magnitude continuously decreases as the current level increases. Previous studies have suggested that in HfO_2_‐based FTJs, two simultaneous switching mechanisms exist.^[^ ^54^, ^55^, ^56^ ^]^ Specifically, at lower *V*_PGM_, FE switching occurs, reducing the *E*‐field within the FE film and increasing the noise. At higher *V*_PGM_, non‐FE switching occurs, similar to the switching mechanism in RRAM, which involves the redistribution of oxygen vacancies within the HZO film, leading to noise reduction. The increase in *S*_IT_/*I*_T_^2^ (*I*_T_ from 20 to 80 nA) as *V*_PGM_ increases indicates current fluctuation due to carrier scattering by FE dipole, while the decrease in *S*_IT_/*I*_T_^2^ (*I*_T_ from 80 nA to 8 µA) suggests current fluctuation driven by a non‐FE mechanism.^[^ ^41^, ^54^, ^55^, ^56^ ^]^ Figure 3B shows the *S*_IT_/*I*_T_^2^ sample at 100 Hz versus *I*_T_. Consistent with Figure 3A, a clear increase, followed by a decrease in *S*_IT_/*I*_T_^2^ with increasing *I*_T_ is observed. The application of PP resulted in a significant reduction in *S*_IT_/*I*_T_^2^ compared with the unpruned case. Figure S9A (Supporting Information) shows *S*_IT_/*I*_T_^2^ when the *I*_T_ is 80 nA (where the noise source is FE switching), demonstrating a substantial reduction (≈1/3) in *S*_IT_/*I*_T_^2^ with the PP application. In contrast, Figure S9B (Supporting Information) shows *S*_IT_/*I*_T_^2^ when the current is 8 µA (where the noise source is non‐FE switching), showing no change in noise size despite PP application. The selective reduction of *S*_IT_/*I*_T_^2^ by PP only in the FE switching‐dominant operation region of the FTJs suggests that the unaligned polarized domains generate additional noise. It appears that the excess noise is caused by the unaligned polarized domains (polarization fluctuations) in the FE‐switching regime of the FTJs. Furthermore, we demonstrate that this excess noise can be significantly reduced by enhancing the alignment of the polarization domain using a PP. Figure 3C shows the mechanism behind the noise generation as electrons pass through the FE layer. As depicted in Figure 3C, the process involves: 1) Electron conduction through the FE layer. 2) Carrier scattering owing to disordered FE dipoles within the FE layer. 3) Fluctuation of the FE dipole as a consequence of carrier scattering. 4) Noise amplification due to additional carrier scattering induced by dipole fluctuations. Consequently, PP significantly contributes to the reduction in noise by mitigating the disordered polarization within the FE layer.

Moreover, the fact that PP reduces *S*_IT_/*I*_T_^2^ in FTJs can be leveraged to decrease the read‐current fluctuations. Figure 3D shows the transient *I*_T_ behavior of FTJs at a current magnitude of 120 nA (top) and 1.2 µA (bottom), over 1 s at a sampling rate of 3200 Hz. Consistent with frequency‐domain measurements (Figure 3B; Figure S9, Supporting Information), the PP reduces the read noise in the time domain, particularly when FE switching is dominant. The read noise in memory devices makes distinguishing between different memory states challenging, thereby limiting the number of usable memory states. By applying PP, more distinguishable memory states are achieved within the same current range, thereby enhancing the memory density. Figure 3E shows that after applying a pruning pulse, the number of distinguishable memory states in the same current range doubled compared to the unpruned case. Figure 3F shows the cumulative probability of the measured transient read (3200 Hz, 1 s) *I*_T_ results in the FTJs, demonstrating improved memory density owing to reduced read noise. The LFN spectroscopy results for the FeFETs are shown in Text 3 (Supporting Information).

### 2.5 Polarization Pruning in Neuromorphic Computing

The improved retention and reduced read noise not only offer significant benefits in memory applications but also offer the potential for advancements in neuromorphic computing.^[^ ^40^ ^]^ Neuromorphic computing can be implemented using two main methods: on‐chip learning, where learning and inference occur within the same hardware,^[^ ^56^ ^]^ and off‐chip learning, where learning is performed using software before transferring weights to the hardware.^[^ ^13^ ^]^ PP demonstrated effectiveness in both approaches.

**Figure 4:**
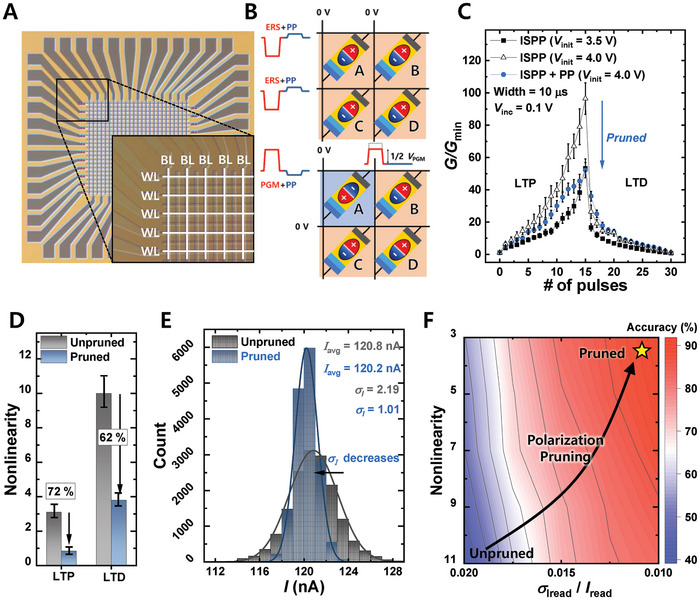
Application of PP in an FTJ array. A) A top optical image of a 23 × 25 FTJ array. B) Operational scheme for utilizing PP within the FTJ array. C) LTP and LTD characteristics of FTJs during weight updates using the ISPP method, comparing scenarios with and without PP. D) Degree of nonlinearity improvement achieved through the application of PP. E) Histogram showing the read noise distribution in the FTJ array, comparing conditions with and without the application of PP. F) Contour graph showing the variation in accuracy of an on‐chip learning FTJ‐based hardware neural network system, influenced by non‐linearity and read variation.

A 23 × 25 FTJ synaptic array was fabricated for on‐chip learning. **Figure** **4**A shows a top‐view optical microscopic image of the fabricated FTJ array. Word lines (WLs) and bit lines (BLs) intersect orthogonally with the FTJs located at junctions. Figure 4B shows a schematic of the operating scheme of the fabricated FTJ array. The detailed operational scheme for the FTJ array (PGM, ERS, inhibition, and PP) is presented in the Experimental Section. Figure S11A (Supporting Information) shows the *I*_T_–*V*_G_ for 100 cells in the FTJ array for different states (PGM and ERS). The average *I*_T_ is represented by a solid line, and the area corresponding to 2*σ* is highlighted with light shading. Figure S11B (Supporting Information) shows the measurement results obtained following the operational scheme in Figure 4B. The results demonstrated that the ERS, selective PGM, and PP functioned correctly in the FTJ array, confirming the successful implementation of the PP scheme at the array level. To demonstrate the effects of PP on on‐chip learning, long‐term potentiation (LTP) and depression (LTD) were investigated with and without PP.^[^ ^57^ ^]^ Ideally, synaptic devices should exhibit consistent conductance changes, regardless of the conductance (*G*) level, in response to potentiation/depression pulses. However, most existing synaptic devices exhibit non‐linearity in which the number of updates varies based on the *G* value. PP was adopted to mitigate this nonlinearity. Figure 4C shows LTP/LTD characteristics obtained from the incremental step pulse program (ISPP) scheme at initial voltages (*V*_init_) of 3.5 and 4.0 V, and also when ISPP (4.0 V) is combined with PP. In the ISPP scheme, the increment in *V*_PGM_ (*V*_inc_) is 0.1 V. Although ISPP allows a high dynamic range, it results in increased non‐linearity as conductance rises. Applying PP can resolve this nonlinearity problem because the amount of FE domain reduction during pruning increases with more programming (Figure 2E). With PP, the required *V*_init_ for the same dynamic range was higher, but non‐linearity was significantly improved, showing improvements of 72% and 62% in LTP and LTD, respectively (Figure 4D). The accuracy of hardware on‐chip learning systems is influenced not only by nonlinearity, but also by read variation. In hardware neuromorphic systems, the vector‐matrix multiplication (VMM) operation sums the currents from each cell according to Kirchhoff's law. Therefore, variations in the read current can affect the accuracy of operations.^[^ ^40^, ^58^ ^]^ As demonstrated earlier (Figure 3), the PP improves the FE dipole alignment and reduces the current fluctuation in the FTJs. Figure 4E shows a distribution of transient current measurement results (5 s, 3200 Hz, 16 000 samples), where current variation (*σ*_I_) is reduced more than twofold by PP. To evaluate the impact of the improved non‐linearity and reduced current fluctuations on the accuracy of hardware‐on‐chip learning systems, a Python‐based simulation was conducted. Figure S12 (Supporting Information) depicts the structure of the VGG11 network used in the simulation.^[^ ^59^ ^]^ Figure S13 (Supporting Information) shows the method for incorporating non‐ideal device factors into the on‐chip learning simulation. See the Experimental Section for the detailed simulation methods. Table S1 (Supporting Information) shows hyperparameters used in on‐chip simulation. Figure 4F shows the accuracy of the hardware on‐chip learning system for the CIFAR‐10 classification. The accuracy improved by over 50% with pruning compared with the unpruned case.

**Figure 5:**
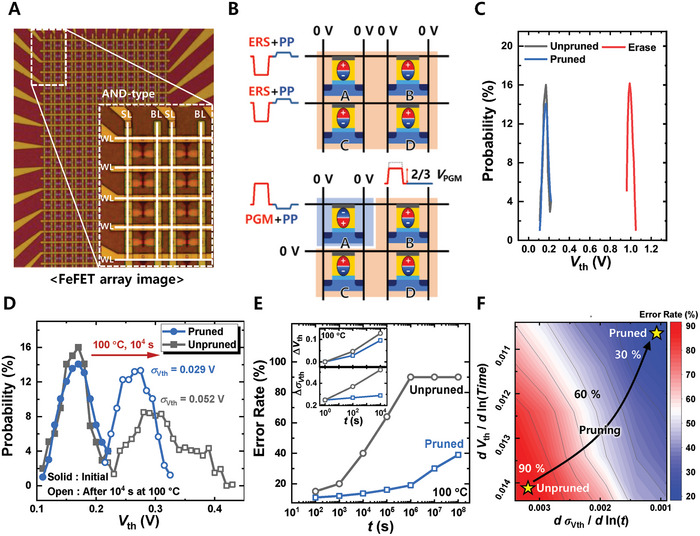
Application of PP in a FeFET array. A) Top optical image of an AND‐type FeFET array. B) Operational scheme for leveraging polarization pruning within the FeFET array. C) *V*_th_ distributions of the FeFET array for three different states: ERS, PGM, and PGM followed by the application of PP. This data illustrate the effect of PP on the *V*th distribution of the FeFET array. D) Changes in *V*_th_ distribution in the FeFET array after 10^4^ s at 100 °C, comparing scenarios with and without PP. E) Variations in the error rate of an off‐chip learning FeFET‐based hardware neural network system over time at 100 °C. The inset provides additional data showing changes in *V*_th_ and *σ_V_*_th_ over time. F) Contour graph showing the variation in accuracy of an off‐chip learning FeFET‐based hardware neural network system, influenced by changes in *V*_th_ and *σ_V_*_th_ over time.

To demonstrate the effectiveness of the PP not only in on‐chip learning but also in off‐chip learning neuromorphic systems, an AND‐type 12 × 24 FeFET array was fabricated, and additional experiments were conducted. **Figure** **5**A shows the top optical microscopy image of the fabricated AND‐type FeFET array. The AND‐type array offers advantages such as a higher density with 8*F* ^2^ (*F*: minimum feature size) compared to 12*F* ^2^ in conventional NOR‐type arrays and the capability for selective programming and erasing, which is not possible in conventional NAND‐type arrays.^[^ ^60^ ^]^ Figure 5B shows a schematic of the operating scheme of the fabricated AND‐type FeFET array. The detailed operational scheme for the FeFET array (PGM, ERS, inhibition, and PP) is presented in the Experimental Section. Figure S14A (Supporting Information) shows the *I*_D_–*V*_GS_ characteristics of the 100 cells in the FeFET array. The average current (*I*_D_) is indicated by a solid line, and the statistical 2*σ* range is represented by lightly shaded areas. Figure S14B (Supporting Information) shows the measurement results following the operational scheme depicted in Figure 5B, demonstrating that the ERS, selective PGM, and PP functioned correctly in the FeFET array, confirming the successful implementation of the PP scheme at the array level. Figure 5C shows the probability distribution of the threshold voltage (*V*_th_) for the ERS, PGM (unpruned), and pruned states. Figure 5D shows the *V*_th_ distribution in a FeFET array after 10^4^ s at 100 °C. The PP not only improves retention characteristics (as indicated by the shift in average *V*_th_) but also mitigates the worsening variation in *V*_th_ (*σ*_Vth_) values over *t*. To evaluate the impact of PP‐induced improvements in retention and *V*_th_ variation on off‐chip learning, a Python‐based simulation is conducted. Figure S15 (Supporting Information) shows the method of incorporating nonideal device factors into the off‐chip learning simulation. The detailed simulation methods are explained in the Experimental Section. Figure 5E shows the *t*‐dependent error rate for the CIFAR‐10 task. As time progressed, the error rate increased, owing to changes in the pretrained weights caused by retention loss. Nevertheless, PP notably mitigates the rate of accuracy degradation compared with the unpruned case. The inset in Figure 5E shows the retention characteristics used in the simulation. Figure 5F illustrates the contour map of error rate in off‐chip learning (after 10^4^ s at 100 °C following post‐pretraining) versus retention characteristics (*dV*_th_/*d*ln(*t*)) and the spread in *V*_th_ over time (*dσ*_Vth_/*d*ln(*t*)). The results clearly show that the rate of error increase is substantially reduced when PP is applied, compared with the unpruned scenario.

**Table** **1** presents a comparison of the various methods proposed to reduce noise in different memory structures. The proposed PP is the first method introduced to reduce noise in FE dipoles. Unlike the PCRAM, which requires changes in the manufacturing process,^[^ ^61^ ^]^ and the RRAM, which requires additional electrical circuits,^[^ ^62^ ^]^ the PP method is significantly simpler and more practical because it requires only the application of a single opposite‐sign pulse. Additionally, it improves the retention, *V*_th_ variation, linearity characteristics. Moreover, this method is versatile and can be applied across various structures, including FeRAM, FTJ, and both 2D and 3D FeFETs, without the need for additional circuitry or changes to the fabrication process.″

**Table 1:** The table summarizes studies reported on methods to reduce current noise in various electrical devices.

Ref	Year	Device type	Methods	Underlying physics	Require unit	Advantage	Note
[61]	2019	PCRAM	Alternately stacking nanolayers to form heterostructres	Confinement nanolayer suppress the noise	Changing fabrication process	‐ Current drift characteristics also improved	First in PCRAM
[62]	2023	RRAM	Denoising process: three level feedback algorithm	Removing incomplete channels	Additional driving circuit	‐ Achieve thousands of conductance levels	First in RRAM
This work	2024	FE‐based memory (FTJ and FeFET)	Polarization pruning:applying an opposite‐sign pulse immediately after PGM/ERS operation	Aligning weakly polarized domain	‐	‐ Retention, *V*_th_ variation, linearity also improved ‐ No additional circuity or fabrication change required ‐ Applicable to various structures (FeRAM, FTJ, and both 2D and 3D FeFET)	First in FE devices

## 3 Conclusion

In this study, we presented the first demonstration of the PP as an operational scheme that enables the alignment of the FE dipole in the desired direction. By applying a weak pulse of the opposite sign to the preceding PGM and ERS pulses, the PP effectively removed the weakly polarized domain. To reveal the underlying physics of the PP, Modified PUND enables quantitative measurement of the amount of pruned polarization and is used to optimize the PP. LFN spectroscopy revealed that the excess noise in the FTJs can be attributed to polarization fluctuations caused by unaligned polarized domains. We demonstrated that PP doubles the conductance levels and improves retention characteristics by more than 20%, significantly enhancing data storage density and stability for neuromorphic computing applications.

Furthermore, beyond verification at the single‐device level, empirical investigations have been extended to the array level, demonstrating the applicability of PP as a practical part of data‐centric operations in neuromorphic computing. PP significantly enhances key factors such as retention, read noise, variability, and linearity in devices for implementing neuromorphic computing. This makes it applicable across different FE‐based memories, including FTJs and FeFETs, and suitable for various neuromorphic computing architectures, both on‐ and off‐chip learning. Ultimately, these findings will contribute to the development of more reliable fluorite FE‐based devices for memory and neuromorphic applications.

## 4 Experimental Section

### Fabrication Process of FTJ and FeFETs

1. FTJ: Prepare an SOI wafer and proceed with *n*‐type implantation. Bottom‐gate patterning was performed by dry etching. After patterning, the wafer was cleaned in the following sequence: SPM solution (H_2_SO_4_: H_2_O_2_ = 4:1, 120 °C, 10 min), APM solution (NH_4_OH: H_2_O_2_: H_2_O = 1: 1: 5, 80 °C, 10 min), HPM solution (HCl: H_2_O_2_: H_2_O = 1: 1: 5, 80 °C, 10 min), and diluted HF solution (HF: H_2_O = 1: 100, 25 °C, 3 min). Subsequently, a 1.2 nm layer of SiO_2_ was deposited using thermal atomic layer deposition (ALD) for eight cycles. Thermal ALD was also used to fabricate the FE HZO layer. The HZO ALD supercycle consisted of two cycles of HfO_2_ and one cycle of ZrO_2_, depositing a 6.23 nm HZO layer over 23 cycles. Finally, a 100 nm TiN layer was deposited via the DC sputtering method, and top‐gate patterning was performed through dry etching. To induce the FE orthorhombic phase in the HZO layer, rapid thermal annealing (RTA) was conducted at 700 °C for 30 s in an N_2_ ambient. For passivation, a 300 nm layer of SiO_2_ was deposited using tetraethyl orthosilicate (TEOS), and contact holes were formed through dry etching. The measurement pads were formed by depositing Ti (30 nm)–TiN (30 nm)–Al(300 nm)–TiN(30 nm) using DC sputtering.
2. FeFET: Prepare a *p*‐type silicon‐on‐insulator (SOI) wafer and define the active area using dry etching. Upon patterning, the wafer was cleaned in the following sequence: SPM solution (H_2_SO_4_: H_2_O_2_ = 4:1, 120 °C, 10 min), APM solution (NH_4_OH: H_2_O_2_: H_2_O = 1:1:5, 80 °C, 10 min), HPM solution (HCl: H_2_O_2_: H_2_O = 1:1:5, 80 °C, 10 min), and diluted HF solution (HF: H_2_O = 1:100, 25 °C, 3 min). Finally, a 1.2 nm SiO_2_ layer was deposited through thermal ALD over eight cycles. Thermal ALD was used to deposit the FE HZO layers. The HZO ALD supercycle comprised two cycles of HfO_2_ and one cycle of ZrO_2_, depositing a 6.23 nm HZO layer through 23 cycles. DC sputtering was used to deposit a 100 nm TiN layer, followed by top‐gate patterning through dry etching. The TiN layer and the corresponding photoresist were used as a mask for ion implantation during source/drain formation, enabling self‐alignment. Ion implantation was performed for source/drain formation using phosphorus, under conditions of 10^15^ cm^−2^ and 10 keV. To induce the FE orthorhombic phase in the HZO layer and activate the dopants in the source/drain, RTA was conducted with N_2_ at 700 °C for 30 s. For passivation, 300 nm SiO_2_ was deposited using TEOS, contact holes were formed, and measurement pads were created by depositing layers of Ti (30 nm), TiN (30 nm), Al (300 nm), and TiN (30 nm) via DC sputtering. Finally, to minimize defects in the FeFET, conduct HPA at 300 °C with a gas mixture of 4% H2 and 96% N_2_ for 30 min.

### Modified PUND Measurement Methods

During the “P” phase, displacement current flows owing to the switching of the FE dipoles by the PGM pulse, as well as leakage and capacitive currents. In the “PP” phase, displacement current stems from the re‐orientation of weakly polarized FE domains, which we denote as “pruning”. During the “PU” phase, the displacement current involves localized switching exclusively for domains reversed during the “PP” phase since polarization switching already occurred in the “P” phase. Capacitive and leakage currents also flowed during this phase. Consequently, the switching current magnitude in the “PU” phase is smaller compared to the “P” phase. In the “U” phase, there are no current components due to switching, only leakage and capacitive currents flow. Thus, integrating the difference in the current measured during the “P” and “U” phases, such as in conventional PUND, refers to the entire polarization switching component. Furthermore, integrating the difference in the current measured during the “PU” and “U” phases quantifies the amount of polarization pruned (reversed) by PP. Therefore, the ratio of the integrated results of “P” and “U” to “PU” and “U” can calculate the proportion of pruned polarization switching relative to the total polarization switching.

### Electrical Measurement

A probe station with a semiconductor parameter analyzer (B1500A) was used to evaluate the synaptic memory properties of the fabricated FeFETs. To measure the PSD of the FeFET, a combination of a Keysight Semiconductor Device Analyzer (B1500A), Stanford Research Systems Low‐Noise Current Preamplifier (SR570), and Keysight Dynamic Signal Analyzer (35670A) was used. Initially, B1500A applied voltage to the gate, followed by SR570, which transformed the variations in the output current into voltage fluctuations. Subsequently, the 35670A interpreted the dynamic signal from the SR570 in the PSD.

### LFN Measurement Methods

Figure S7 (Supporting Information) outlines the proposed measurement scheme for analyzing the impact of PP on the LFN characteristics of the FE devices. A varying *V*_PGM_ was applied to the top gate of the FE device to measure the LFN in different PGM states. Upon applying the PP pulse, a constant read bias was applied to the electrode and this state was maintained for 5 s (Figure S7A, Supporting Information). This step is essential to ensure that the current stabilizes without any drift after the application of the program and PP pulses. This is because any drift in the current can overshadow other noise components, making accurate LFN measurements challenging. Once the current drift stabilized, the current fluctuations were measured and a Fourier transformation was conducted to calculate the PSD in the frequency domain (Figure S7B, Supporting Information). Detailed information on the measurement equipment, their roles, and the configuration of the devices and equipment are shown in Figure S8A (Supporting Information). Figure S8B (Supporting Information) shows the baseline noise of the measurement system. The measured results are several orders of magnitude higher than those of the noise floor, which confirms that the observed noise is inherent to the device and does not originate from the measurement system. Figure S8C (Supporting Information) shows ten times of LFN measurement results for a single device, demonstrating the consistency of each measurement, thereby confirming the reliability of the measurement.

### Operational Schemes for the FTJ Array: PGM, ERS, Inhibition, and PP

Initially, to erase all cells, ERS and PP pulses were applied to the WLs, whereas 0 V was applied to the BLs. To program the cells selectively, PGM and PP pulses were applied to the WL of the cell to be programmed, with 0 V applied to the other WLs. Note that Figure 4b shows the case of the selectively programmed cell A. In cells on the selected WL that were not targeted for programming, an inhibition voltage of half the magnitude of the PGM pulse was applied to the BL to prevent unintentional programming. Considering the relatively low amplitude of the PP pulse, a separate inhibition pulse was unnecessary.

### Operational Schemes for the FeFET Array: PGM, ERS, Inhibition, and PP

The process begins by erasing all cells by applying ERS and PP pulses to the WLs, with 0 V applied to both the BLs and source lines (SLs). For selective programming (assuming the programming of cell A in Figure 5B), PGM and PP pulses were applied to the WL of the targeted cell, whereas 0 V was applied to other WLs. For the cells on the selected WL that were inhibited, inhibition pulses were applied to both the BL and SL. An inhibition pulse was set at two‐thirds the magnitude of the PGM pulse, ensuring that unintended programming was prevented. Given the small amplitude of the PP pulse, an additional inhibition pulse during the PP is not required.

### Hardware Neuromorphic Simulation Methods

1. On‐chip learning: Figure S11 (Supporting Information) shows the on‐chip learning simulation process used in the experiment. Weight initialization is performed randomly, followed by forward propagation of the input test dataset. In neuromorphic systems, VMM is performed based on Kirchhoff's law, and the result is represented as a current (or integrated charge). Therefore, read current noise affects the accuracy of the network. Consequently, the read noise measurement results shown in Figure 3F were incorporated into the simulation. Subsequently, backpropagation was conducted based on the error between the output and the correct labels. Backpropagation also passes through the transposed weight matrix; thus, it is influenced by read noise. Weight updates were then conducted based on the values obtained from the backpropagation, with the measured nonlinearity from Figure 4C reflected during the process. At the end of 100 learning epochs, the maximum accuracy was evaluated.
2. Off‐chip learning: Figure S13 (Supporting Information) shows the off‐chip learning simulation process used in the experiment. Off‐chip learning is conducted by transferring pre‐trained weights from a software‐based platform. Therefore, pre‐trained weights that demonstrated a 91.8% accuracy for the CIFAR‐10 dataset were transferred. Since the weight transfer may not be precise, owing to the device‐to‐device (D2D) variation in the FeFETs, the measured D2D variation (Figure S12, Supporting Information), was incorporated into the transfer process. The performance degradation of the off‐chip learning system over time was assessed. Each of the transferred weights tended to drift over time. To account for these retention characteristics, the measurement results were utilized. The inset of Figure 5E shows the shift in average *V*_th_ (Δ*V*_th_) and changes in sigma of *V*_th_ (Δ*σ*_Vth_) measured from 100 cells. Based on these time‐dependent values, alterations were made to the weight matrix. The output accuracy of the neural network with the altered weight matrix was evaluated using the test dataset.

## Conflict of Interest

The authors declare no conflict of interest.

## Supporting information



Supporting Information

## Data Availability

The data that support the findings of this study are available from the corresponding author upon reasonable request.
