# Supplementary material for: Polarization Pruning: Reliability Enhancement of Hafnia‐Based Ferroelectric Devices for Memory and Neuromorphic Computing
Source: Adv Sci (Weinh). 2024 Sep 26;11(43):2407729. doi: 10.1002/advs.202407729 (PMC11578341; doi:10.1002/advs.202407729)
Supplement: Supplementary file 1 — Supporting Information [file ADVS-11-2407729-s001.docx]

Supplementary Materials for

**Polarization Pruning: Reliability Enhancement of Hafnium-based Ferroelectric Devices for Memory and Neuromorphic Computing**

Ryun-Han Koo *et al.*

*Corresponding author. Email: jhl@snu.ac.kr

**This PDF file includes:**

Supplementary Text 1 to 3

Figs. S1 to S15

Supplementary Table 1

Supplementary Text

**1. Ratio of pruned polarization according to the magnitude and sign of *V*_PP_**

Fig. S3D shows the ratio of the pruned polarization according to the magnitude and sign of *V*_PP_. When the absolute values of *V*_PP_ are 2.0 and 2.5 V, there is a significant difference in ∆*P*_PP_ values depending on the sign of *V*_PP_. This discrepancy arises because of the asymmetrical *E*_c_ of the fabricated MFIS FTJ (Fig. S2D). When the absolute values of *V*_PP_ are 2.0 and 2.5 V, switching occurs that reverses well-aligned polarization domains, and the difference in *E*_c_ for positive and negative voltages leads to variations in ∆*P*_PP_ values depending on the sign of *V*_PP_. Conversely, when the absolute values of *V*_PP_ are 0.5 and 1.0 V, there is no difference in ∆*P*_PP_ values based on the sign of *V*_PP_. This is because the switching induced by the *V*_PP_ pulse is unrelated to *E*_c_ and originates from reversing the weakly polarized domains that are not fully aligned in the original program state. Thus, these results provide further evidence supporting our claim regarding the principle of PP and theoretically demonstrate its applicability to both the PGM and ERS states.

**2. Endurance characteristics of FE-based memory with PP**

Fig. S6 shows the endurance characteristics of the FE film. Fig. S6A shows the *P*_r_ values of the HZO layer subjected to PGM/ERS cycling stress (PE cycle) at different temperatures (*T*=20, 60, and 100 °C). The case without applying PP during the PE cycle is denoted as ‘Unpruned’, while the case with PP during the PE cycle is denoted as ‘Pruned’. It's worth noting that, for a fair comparison, the memory window conditions are consistent regardless of PP application (thus, ~0.1 V lower PGM and ERS voltages are utilized for cases without PP). Across all temperature ranges, no significant difference was observed in the *P*_r_ degradation trend between the cases with and without PP. Fig. S6B presents the Arrhenius plot of *P*_r_ degradation with and without PP, indicating that the activation energy (*E*_a_) of degradation does not significantly differ with the presence of PP (*66*). Fig. S6C shows the interface trap density (*D*_it_) extracted by multi-frequency impedance spectroscopy (*67*) for three states of FTJ: the pristine state (Prs), the state with 10^6^ cycling stress applied without PP (Un.P), and the state with 10^6^ cycling stress applied alongside PP pulses (P). After applying the PE cycling stress, an increase in *D*_it_ by 37-44 % was observed, with minimal difference due to the presence or absence of PP. From these results, it is evident that the presence of PP pulses does not significantly impact the endurance characteristics of the FE thin layer. Considering the trade-off relationship between endurance and retention characteristics commonly observed in memory devices, PP, which can dramatically improve retention characteristics without compromising endurance, offers substantial advantages.

**3. FeFET low-frequency noise analysis results**

Unlike FTJs where the path of current flow overlaps with the polarization switching area, in FeFETs, the current flows through the silicon channel located between the source and drain. This channel is separate from the area where polarization switching occurs. Fig. S10A presents a graph showing the normalized PSD of *I*_D_ (*S*_ID_/*I*_D_^2^) versus frequency for different *V*_GS_s applied to a FeFET. Across all operational ranges, a 1/*f* noise trend is observed. As *V*_GS_ increases (corresponding to an increase in drain current, *I*_D_), the magnitude of *S*_ID_/*I*_D_^2^ decreases. Fig. S10B shows the results of measuring transient drain current in various operation regions (20 nA, 200 nA, 2 μA) depending on the application of PP, measured over 5 seconds at 3200Hz. Unlike FTJs, no change in current fluctuation magnitude is observed depending on the presence or absence of PP in FeFETs. To understand the noise generation mechanism in FeFETs, *S*_ID_/*I*_D_^2^ is measured across various *I*_D_s and plotted in Fig. S10C, sampled at 100 Hz. The open symbols in fig. S10C represent *S*_ID_/*I*_D_^2^ measured at each *I*_D_ value, while the solid lines correspond to (*g*_m_/*I*_D_)^2^ × *k* (*g*_m_ : transconductance of FeFET, *k* : fitting parameter). When the noise generation mechanism is carrier number fluctuation (CNF), the following relationship holds (*68-70*):

$$\boldsymbol{S}_{\mathbf{ID}}\mathbf{=}\boldsymbol{S}_{\mathbf{Vfb}}\boldsymbol{\times}{\boldsymbol{g}_{\mathbf{m}}}^{\boldsymbol{2}}$$

$$\boldsymbol{S}_{\mathbf{vfb}}\mathbf{=}\frac{\boldsymbol{q}^{\boldsymbol{2}}\boldsymbol{kT\lambda}\boldsymbol{N}_{\boldsymbol{t}}}{\boldsymbol{f}^{\boldsymbol{\gamma}}\boldsymbol{WL}{\boldsymbol{C}_{\boldsymbol{ox}}}^{\boldsymbol{2}}}$$

where *S*_Vfb_ is the flat-band voltage PSD, *C*_ox_ is the oxide capacitance, *f* is frequency, *W* is the width of the FeFET, *L* is the length of the FeFET, *N*_t_ is the density of traps at the quasi-Fermi level, $\boldsymbol{\lambda}$ is the tunneling attenuation length in the gate oxide. *k* is the Boltzmann’s constant and *T* the is temperature.
 Therefore, the correspondence between *S*_ID_/*I*_D_^2^ and (*g*_m_/*I*_D_)^2^ trends in Fig. S10C, regardless of PP, indicates that the noise generation mechanism in FeFETs is attributed to CNF. The source of CNF is the trapping-detrapping of electrons passing through the Si channel at the interface with the dielectric. Therefore, the absence of significant change in the normalized PSD of FeFETs after applying the PP pulse (Fig. S10C) further confirms that PP primarily affects dipole alignment within the ferroelectric layer without altering the electron trapping-detrapping processes in the Si channel. This result supports our hypothesis that the effects of PP originate from dipole alignment, as evidenced by the observed noise reduction in FTJs but not in FeFETs. Fig. S10D provides a schematic diagram of the *I*_D_ noise generation mechanism in FeFETs.

Fig. S1. Schematic diagram of the FTJ fabrication process. (A) An SOI wafer is prepared followed by *n*-type doping via ion implantation. (B) Bottom gate patterning using anisotropic etching. (C) Deposit a 1.2 nm layer of SiO_2_ through chemical oxidation and a 6.4 nm layer of HZO using thermal ALD. (D) Deposit a 100 nm layer of TiN using DC sputtering. (E) Top gate patterning using anisotropic etching, followed by the formation of pads through back-end processes.

Fig. S2. Material and electrical characteristics of the FTJ. (A) Cross-sectional TEM image of the MFIS-FTJ. (B) XPS analysis results showing the composition of HZO layer. (C) GIXRD patterns of HZO before and after annealing, with orthorhombic peaks emerging post-annealing. (D) PUND measurement results. (E) *P*-*V* and (F) *I*_T_-*V* curves of the fabricated MFIS FTJ.

Fig. S3. Measurement results for analyzing the underlying mechanism of polarization pruning. (A) Relationship between pruned polarization and *W*_PP_ for various *V*_PP_ values, without the previous *V*_PGM_ pulse. (B) Variation in pruned polarization as a function of the number of applied pruning pulses (*N*_PP_). Consistent with observations in Fig. 2B, a saturation trend in the increase of pruned polarization is seen under both underpruned and optimally pruned scenarios when *N*_PP_ is varied, in contrast to the overpruned state where no saturation occurs. (C) Time-dependent retention characteristics of the FTJ at 20 ℃. An improvement in retention characteristics is observed with pruning, while overpruned cases result in worsened retention. (D) Pruned polarization dependent on the amplitude of *V*_PP_. (E) Time-dependent polarization changes in FTJ. The graph compares the retention characteristics of pruned versus unpruned cases. (F) Dependency of pruned polarization on the size of the FTJ. (G) Correlation between the size of the FTJ and the retention improvement achieved through polarization pruning.

Fig. S4. Schematic diagram of the FeFET fabrication process. (A) SOI wafer preparation. (B) Define active regions through anisotropic etching. (C) Deposit a 1.20 nm SiO_2_ layer through chemical oxidation, followed by the deposition of a 6.23 nm HZO layer using thermal ALD. (D) Apply DC sputtering to deposit a 100 nm TiN layer. (E) Define top gate through anisotropic etching. (F) Form source/drain area via ion implantation, followed by post-metal annealing to form the FE o-phase in the HZO layer. Perform high-pressure annealing to reduce trap sites, and then proceed with the back-end process. (G) Flow chart of the fabrication process. (H) Cross-sectional TEM image of the fabricated FeFET

Fig. S5. FTJ retention characteristics with and without Polarization Pruning. (A) The retention characteristics of FTJs over time at different temperatures (20, 60, and 100 °C), comparing the effects of applying polarization pruning. (B) The change in Δ*P*_r_ in FTJ over 1000 seconds at various temperatures, highlighting the impact of PP application on polarization retention.

Fig. S6. Endurance characteristics of the FE thin film depending on the application of PP. (A) Endurance characteristics of the FTJ at various temperatures. (B) Extracted *E*_a_ during the degradation process. (C) Graph representing the *D*_it_ for the pristine state (Prs.), the state after 10^6^ updates without PP application (Un.P.), and the state after 10^6^ updates with pruning application (P.). (D) Changes in the MW of FeFETs due to repeated application of the pruning pulse. The amplitude of the pruning pulse is much smaller than that of the program or erase pulses, indicating that it has minimal impact on the endurance characteristics of the FeFET.

Fig. S7. Methodology for low-frequency noise (LFN) spectroscopy measurement. (A) Pulse scheme utilized in the LFN spectroscopy process. (B) Schematic diagram illustrating the process of converting the measured current from step (A) into PSD.

Fig. S8. LFN Spectroscopy Measurement Environment. (A) Configuration of the LFN measurement system's equipment used in this experiment. (B) Noise floor of the measurement system during the experiment. (C) Results of ten LFN measurements (light blue) and the averaged PSD (blue).

Fig. S9. Changes in normalized PSD depending on PP application at different current levels in FTJs. (A) At a current level of 80 nA, and (B) at a current level of 8 μA, the variation in *S*_IG_/*I*_G_^2^ versus frequency depending on whether PP is applied.

Fig. S10. Low-frequency noise (LFN) spectroscopy in FeFETs. (A) *S*_ID_/*I*_D_^2^ versus frequency at different *I*_D_ levels. (B) Results of measuring the transient *I*_D_ in FeFETs. Unlike in FTJs, the presence or absence of PP does not affect current variation in FeFETs. (C) Correlation of *S*_ID_/*I*_D_^2^ with (*g*_m_/*I*_D_)^2^, and *I*_D_. The observed trends in *S*_ID_/*I*_D_^2^ and *g*_m_/*I*_D_^2^ relative to *I*_D_ are consistent, indicating that the noise generation mechanism of FeFET is CNF, and the presence of PP does not impact the magnitude of *S*_ID_/*I*_D_^2^. (D) Schematic diagram illustrating the noise generation mechanism in FeFETs (CNF).

Fig. S11. Measurement results of the FTJ array. (A) *I*_G_-*V*_G_ measurement results for the FTJ. The lightly shaded area represents the statistical 2*σ* range. (B) Changes in the *V*_th_ of each cell during updates, following the operation scheme shown in Fig. 5B.

Fig. S12. Structure of the VGG11 network used in simulation. The network architecture for feature extraction includes 5 max-pooling layers and 8 convolution layers. For classification, 3 fully connected layers are utilized.

Fig. S13. On-chip learning process flow chart. The chart details the on-chip learning process implemented in the simulation, emphasizing the integration of device non-ideal factors accounted for during the simulation process.

Fig. S14. Measurement results of the FeFET array. (A) *I*_D_-*V*_GS_ measurement results for the FeFET, with the lightly shaded area representing the statistical 2*σ* range. (B) Changes in the *V*_th_ of each cell during updates, following the operation scheme shown in Fig. 5B.

Fig. S15. Off-chip learning process flow chart. The chart details the off-chip learning process implemented in the simulation, emphasizing the integration of device non-ideal factors accounted for during the simulation process.


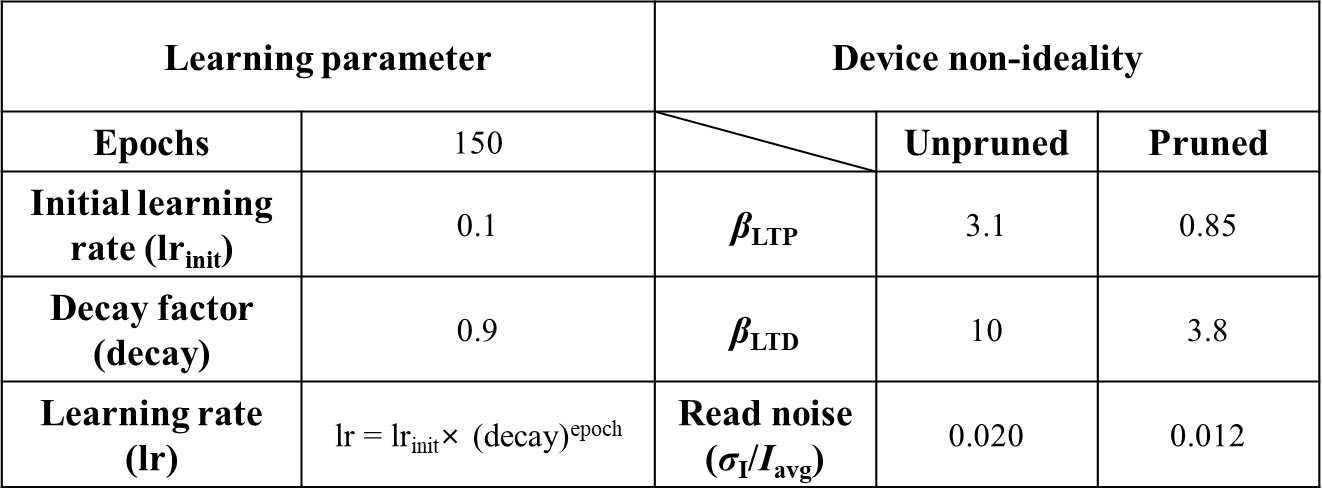


Table. S1. Hyperparameters used in on-chip simulation. The table lists the hyperparameters used in the on-chip simulation, including the number of epochs, initial learning rate, decay factor, learning rate, nonlinearity of LTP and LTD with and without the application of pruning pulses, and read noise with and without the application of pruning pulses.
